# Evaluation of the Secretor Status of ABO Blood Group Antigens in Saliva using Absorption Inhibition Method

**DOI:** 10.1055/s-0041-1723083

**Published:** 2021-02-23

**Authors:** M.L Avinash Tejasvi, Jaya Laksmi Bukkya, Pandu Ranga Rao, Harsha Bhayya

**Affiliations:** 1 Kids Research, Nalgonda, Telangana, India; 2 Department of Oral Medicine and Radiology, Tirumala Dental College, Nizamababad, Telangana, India; 3 Department of Transfusion Medicine, Kamineni Institute of Medical Sciences, Narketpally, Telangana, India; 4 Department of Oral Medicine and Radiology, HKDET Dental College and Hospital Humnabad, Karnataka, India

**Keywords:** absorption inhibition method, slide agglutination method, secretors

## Abstract

**Objectives** While DNA profiling has become the principal technique for individualization of biological evidences, ABO blood grouping is still a useful test method in the initial stages of crime investigation. Objectives of the study were blood group determination using slide agglutination method, blood group determination from saliva using absorption inhibition method, and comparison of slide agglutination method with that of absorption inhibition method from saliva sample.

**Materials and Methods** A total of 60 subjects were taken randomly with their age ranging from 20 to 60 years. Sixty subjects were divided in to two groups, study group and control group. 5 to 10 mL of unstimulated saliva was collected from 60 patients and Wieners agglutination test was performed to detect the secretor status of blood using absorption inhibition method and compared with that of slide agglutination method

**Results** Out of 60 subjects, 52 subjects showed secretors of antigen in saliva with percentage value of 86.66% and eight subjects were nonsecretors (13.33%). Slightly higher percentage of secretor status was seen in males 84.6 and 88.2% in females.

**Conclusion** Evaluation of secretor status of blood group antigen from saliva using absorption inhibition method can be useful method in identification of medicolegal cases.

## Introduction

The term “blood group” refers to the entire blood group system which contains red blood cell antigens which are specifically controlled by a series of genes which can be linked closely to the same chromosome or can be allelic. In the year 1990 discovery of ABO blood group system was done by Karl Landsteiner for which he was awarded a noble prize in 1930. 1

There are 33 blood group systems listed by International Society of Blood Transfusion which represent over 300 antigens. Of all the 33 blood group systems, ABO remains the important blood group system because of the fact that anti-A and anti-B antibodies are more prominent clinically 6 months after birth. H antigen is the precursor of the ABO blood group antigen. 2

In day-to-day life, biological evidence is very important in the medicolegal cases. Blood is considered as one of the important evidence in crime scenes and disasters because once the blood group is determined it is unchanged throughout the life. Apart from blood, antigens are also secreted from various body secretions like saliva, nasal secretion, semen, tears, urine, sweat, etc. Blood grouping depends on the secretor status whether the person is a secretor or nonsecretor which is determined by the presence of Lewis antigen. 3 There are two methods for detection of ABO blood group from saliva; they are absorption inhibition method and absorption-elution method. 4

The present study was conducted for the assessment of ABH secretor status from saliva by absorption inhibition method.

## Materials and Methods

A cross sectional study was conducted in the department of oral medicine and radiology in collaboration with transfusion medicine. A total of 60 healthy individuals' age ranged between 20 and 60 years were taken on random basis as there was no exclusion criterion. After obtaining the ethical clearance from Institutional Ethical Committee Board patients were explained in detail about the study, and informed consent was obtained. Sixty patients were divided into two groups; slide agglutination method considered as control group and absorption inhibition method as study group.

### ABO Blood Grouping from Blood

**Fig. 1:**
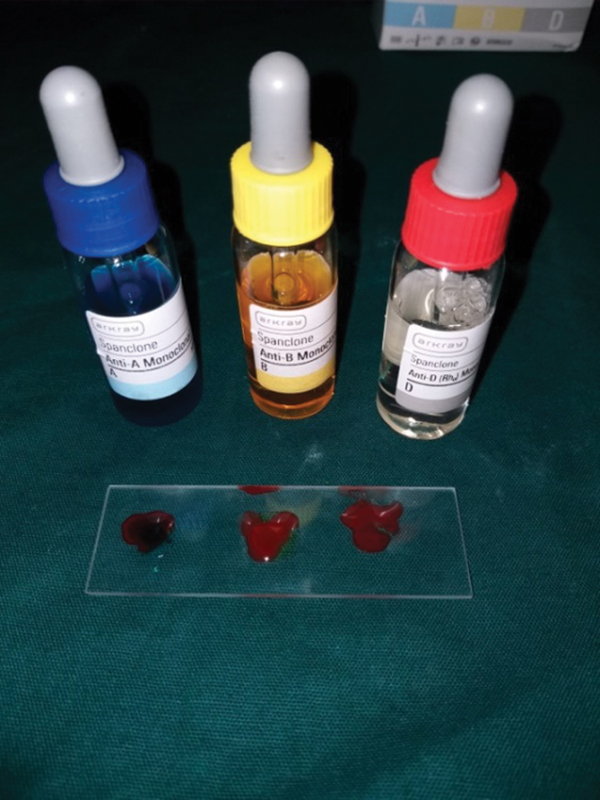
Antisera kit.

Capillary blood was drawn from the patient by a prick on the ring finger, using slide agglutination method. ABO blood grouping was determined in all the patients. A drop of blood was placed on two slides respectively and then antisera A and antisera B were added to each slide and mixed properly later viewed for agglutination as shown in Fig. 1 . The results were recorded; blood group determination using slide agglutination method is considered as a gold standard against which salivary blood group was compared.

### ABO Blood Grouping from Saliva-Absorption Inhibition Method

Reagents used are:

1. Anti-A, anti-B (polyclonal)
2. Anti-H
3. Anti le ^a^
4. A _1_ and B cells
5. Group O, Le (a + b − )cells

**Fig. 2:**
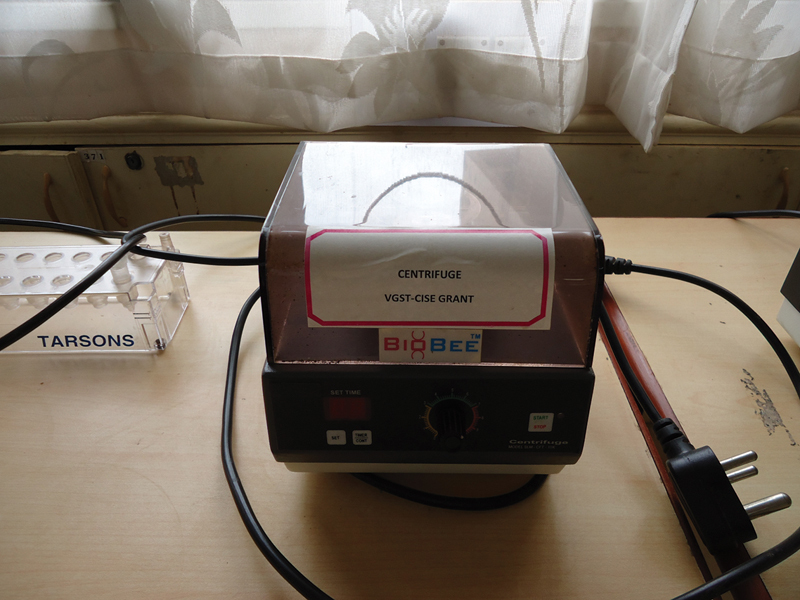
Centrifuge.

**Fig. 3:**
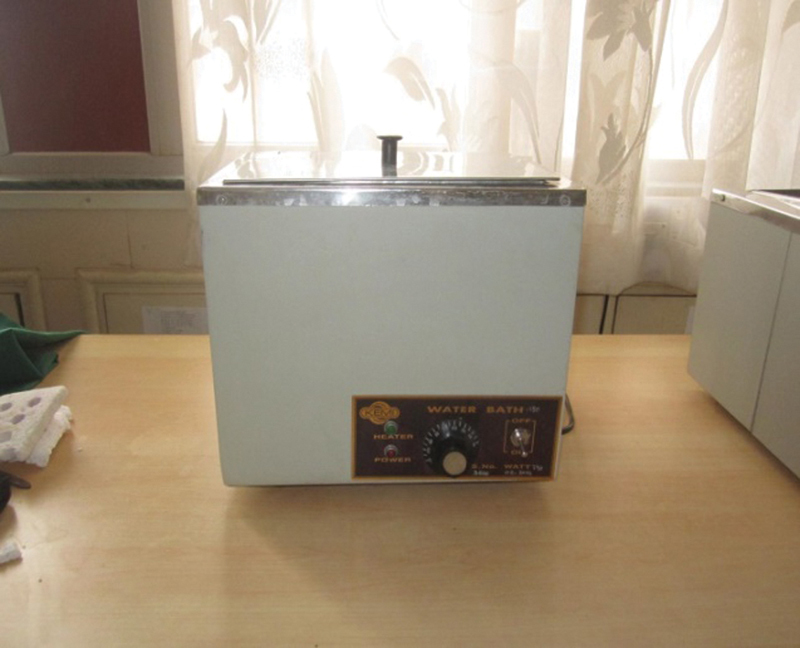
Water bath.

*Specimen collection* : Five to 10 mL of unstimulated saliva was collected from the subjects using spitting method. Saliva was transferred into sterile test tube, centrifuged at 1,700 rpm for 8 to 10 minutes as shown in Fig. 2 and placed in a boiling water for 10 minutes ( Fig. 3 ); later supernatant was separated and recentrifuged at 1,700 rpm for 8 to 10 minutes and diluted with equal volume of saline and stored at −20°C temperature.

### Blood Grouping Reagent Dilution

**Fig. 4:**
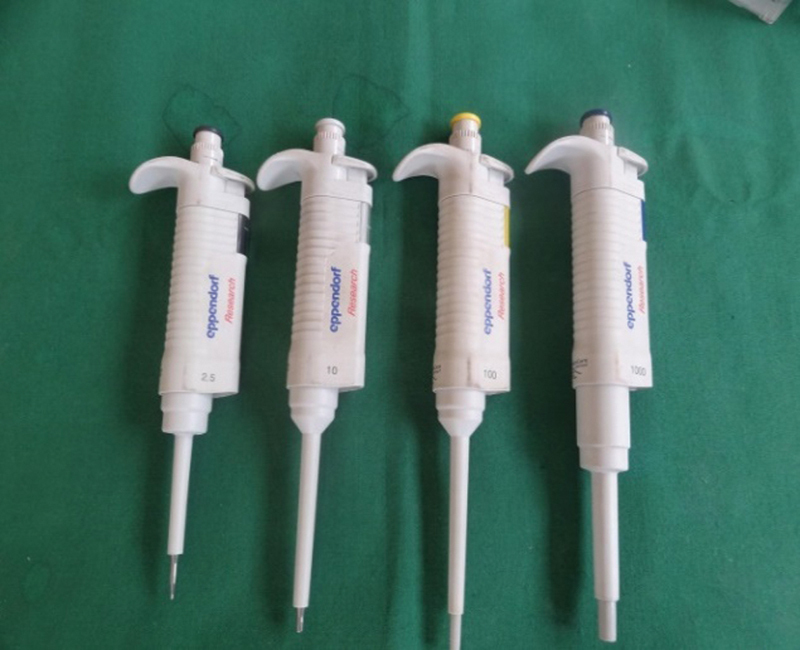
Micropipettes.

*Procedure* : Twelve test tubes were taken, and labeling was done. In these test tubes 200 mL of saliva was added with micropipettes ( Fig. 4 ) and three below mentioned were followed

Step 1: Antisera-A was added in one to four test tubes and dilution was done till 1:16.
Step 2: Antisera-B was added in five to eight test tubes and dilution was done till 1:16.
Step 3: Antisera-D was added in nine to 12 test tubes and dilution was done till 1:16.

After dilution was done, one drop of freshly prepared 2 to 5% of pooled cells of A,B,O blood group were added separately in the test tubes, centrifuged later and checked for the highest reagent dilution. Then the antisera with the highest dilution was used for the inhibition test to know the secretor status.

### Inhibition Test for Secretor Status

To know the secretor status another four test tubes were taken, and the following procedure was followed.

In test tube1 one drop of saliva and one drop of reagent (antisera-A) were added; in test tube 2 one drop of saliva and one drop of reagent (antisera-B) were added; in test tube 3 one drop of saline and one drop of reagent (antisera-A) were added; in test tube 4 one drop of saline and one drop of reagent (antisera-B) were added and incubated for 8 to 10 minutes one drop of 2 to 5% suspension (A,B,O) cells were added and incubated for 30 to 60 minutes, then centrifuged and checked for the highest clumping in the test tube 4.

**Fig. 5:**
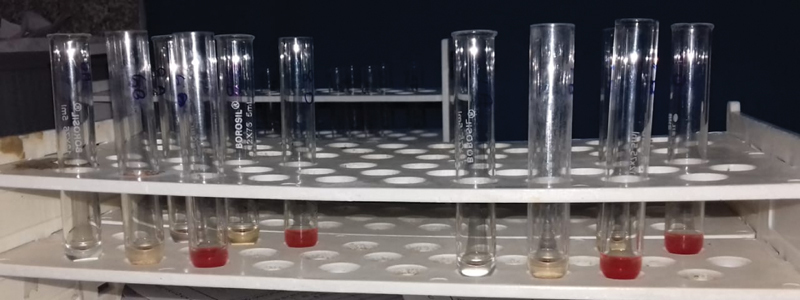
Saliva samples after inhibition test.

**Fig. 6:**
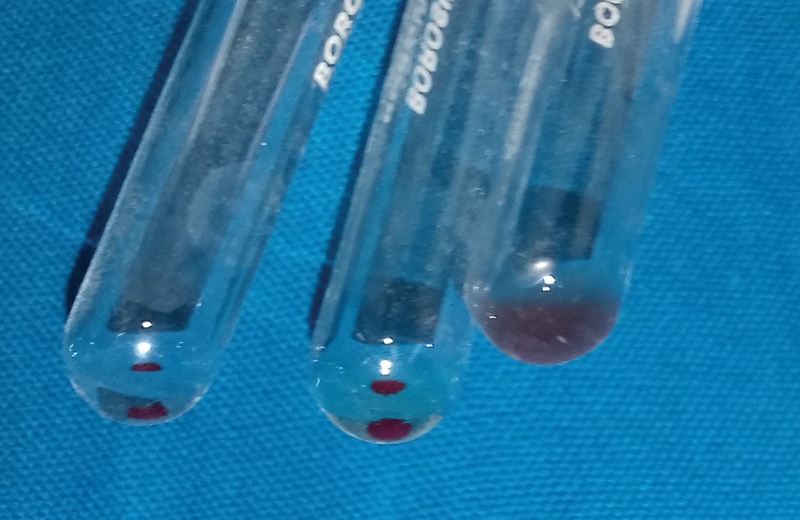
Saliva samples after inhibition test.

*Observations* : All study samples showed clumping in slide agglutination method as there was no antigen. The test group was considered as positive if agglutination was not seen, indicating the presence of blood group ( Figs. 5 and 6 ).

## Results

Out of 60 subjects, 26 (43.33%) were males and 34 (56.66%)were females ( Table 1 ). In the present study 52 subjects showed secretors of antigen in saliva with percentage value of 86.66% and eight subjects were nonsecretors (13.33%) of which four were females and four were males ( Table 2 ). A slightly higher percentage of secretor status is seen in males, i.e., 84.6 and 88.2% in females.

**Table 1:** Demographic data

S. no.	Male	Percentage	Female	Percentage
Slide agglutination method (control group)	26	43.33%	34	56.66%
Absorption inhibition method (study group)	26	43.33%	34	56.66%

**Table 2:** Determination of secretor status from saliva using absorption inhibition method

Group	Secretor status			
	Secretor	Percentage	Nonsecretor	Percentage
Absorption inhibition method ( *N* = 60)	52	86.66%	8	13.33%

### Distribution of Blood Groups in Case and Control Group

*Control group* (slide agglutination method): of 60 subjects, 30 subjects showed O blood group, 16 subjects showed B blood group, 10 subjects showed A blood group, and four subjects showed AB blood group. *Study group* (absorption inhibition method): of 60 subjects in which 26 subjects showed O blood group, 14 subjects showed B blood group, eight subjects showed A blood group, and four subjects showed AB blood group, respectively ( Table 3 ).

**Table 3:** Shows distribution of blood groups in case and control group

S. no.	Blood group	Blood group determination		Percentage
		Blood	Saliva	
1	O	30	26	86.66%
2	B	16	14	87.5%
3	A	10	8	80%
4	AB	4	4	100%

When compared with slide agglutination method and absorption inhibition method 30 subjects showed O blood group in slide agglutination method and 26 subjects showed O blood group in absorption inhibition method with sensitivity of 86.66%. Sixteen subjects showed B blood group in slide agglutination method and 14 subjects showed O blood group in absorption inhibition method with sensitivity of 87.5%.

Twenty subjects showed A blood group in slide agglutination method and 16 subjects showed A blood group in absorption inhibition method with sensitivity of 80%. Four subjects showed AB blood group in slide agglutination method and four subjects showed AB blood group in absorption inhibition method with sensitivity of 100%.

Of all the blood groups highest percentage is seen in AB blood group in both slide agglutination and absorption inhibition method.

### Scoring of Results

4+ complete: one complete mass of agglutinates seen.
3+ visual: large separate mass of agglutinates seen.
2+ double plus: small agglutinates seen.
1+ A granular appearance seen.

Weak small clumps of four to six cells were observed in microscope.

## Discussion

Identification of blood group especially from saliva has been important in establishing the identification of criminals, victims in crime scenes. 4 In the year 1928 presence of anti-A and anti-B hemagglutinins was first analyzed in saliva. Because of inadequate procedures it was not in use. 5

The most common method of blood grouping is from a drop of blood using finger prick method. Apart from blood, antigens are also secreted from various body secretions like tears, sweat, saliva, urine, gastric juice, semen, nasal secretions, etc. 3 Saliva and other body fluids contain ABO antigens which are under control of secretory gene. 6 The ability to secrete blood group antigens in saliva is called as “secretors.” The lack of ability to secrete blood group antigens in saliva is called “nonsecretors.” 7

Secretor status is determined genetically based on the pair of allomorphic genes: Se and se .Se is dominant over se gene. Individual is considered homozygous with sequence of Se-Se. Individual is considered heterozygous with sequence of Se-se. Individual with se-se sequence is considered as nonsecretors. Se is the dominant allele which regulates expression of H-tranferase. 8 The present study showed eight nonsecretors because of their inability to secrete blood group antigen in saliva.

A study was conducted by Emeribe and Igweagu to detect the presence of ABH antigen in saliva using absorption—inhibition method in 176 subjects and results showed 84.90% secretors. The results of the present study showed similar results with a slight difference in the secretor status of 86.66%. 9

Motghare et al conducted a study in 200 subjects to detect secretor status from saliva using absorption inhibition method and results showed 83% were secretors which was similar to that of present study. 3

A study conducted by Bakhtiari et al in 30 subjects to detect a relationship between secretor status and lichen planus using absorption–inhibition method and results showed 84.44% were secretors and 16.6% were nonsecretors. The results of the present study showed 86.66% were secretors. 10

Metgud et al conducted a study to determine secretor status of blood group antigen in saliva. Eighty patients were studied among which 100% were secretors for A and O blood group and 95% were secretors for A and AB blood groups, respectively. 11

Al-Sihli AA conducted a study in 30 subjects to assess the correlation between blood groups and dental caries using absorption–inhibition method and results showed 63.3% were secretors and 36.7% were nonsecretors. Present study shower more secretor status. 12

## Conclusion

In the present study, 86.66% showed secretor status. Absorption inhibition method is a better method in the determination of secretor status from saliva. This method may be helpful in identification of individual in medicolegal cases.
